# Pain and quality of life in breast cancer patients

**DOI:** 10.6061/clinics/2017(12)07

**Published:** 2017-12

**Authors:** Weruska Alcoforado Costa, Michelly Nóbrega Monteiro, Janice França Queiroz, Ana Katherine Gonçalves

**Affiliations:** Universidade Federal do Rio Grande do Norte, Natal, RN, BR

**Keywords:** Breast Cancer, Quality of Life, Health-Related Quality of Life, Pain, Short McGill Pain Questionnaire

## Abstract

**OBJECTIVE:**

To evaluate the influence of pain on quality of life in breast cancer patients.

**METHODS:**

A cross-sectional study of 400 patients, including 118 without metastasis, 160 with loco-regional metastasis and 122 with distant metastasis. The instruments used were the European Organization for Research and Treatment for Cancer Quality of Life Questionnaire-Core 30 and the Breast Cancer-specific 23 and short McGill Pain Questionnaire.

**RESULTS:**

In total, 71.7% of patients reported pain. The most frequent sensory descriptor used by patients was ‘jumping.’ In the evaluative dimension, the main descriptor chosen was troublesome. The Global Health self-assessment showed pain to be inversely correlated with quality of life: the group without metastasis had a mean score of 55.3 (SD=24.8) for those in pain, which rose to 69.7 (SD=19.2) for those without pain (*p*=0.001). Subjects with loco-regional metastasis had score of 59.1 (SD=21.3) when in pain, and those without pain had a significantly higher score of 72.4 (SD=18.6) (*p*<0.001). Patients from the distant metastasis group showed similar results with a mean score of 48.6 (SD=23.1) for those in pain and 67.6 (SD=20.4) for those without pain (*p*=0.002). Regarding the association of pain intensity and quality of life, patients with distant metastasis and intense pain had the worst scores for quality of life with a functional scale mean of 49.9 (SD=17.3) (*p*<0.009), a Symptom Scale score of 50.0 (SD=20.1) (*p*<0.001) and a Global Health Scale score of 39.7 (SD=24.7) (*p*<0.006).

**CONCLUSIONS:**

Pain compromises the quality of life of patients with breast cancer, particularly those with advanced stages of the disease.

## INTRODUCTION

Breast cancer (BC) is often associated with long-term psychological distress, chronic pain, fatigue and impaired quality of life (QoL) 1-5. Approximately 20% to 50% of patients complain about pain, a number which rises to 90% for patients in metastatic or terminal stages 6-10.

Pain is one of the most frequently reported adverse effects that occurs as part of the disease process or as a side-effect of treatment. It is a problem for a majority of BC patients and has an unfavorable effect on QoL 11-15.

More recent epidemiological data obtained from a meta-analysis suggest that pain is prevalent in 39.3% of cases after curative treatment, 55.0% during cancer treatment, and in 66.4% in advanced or terminal stages of the disease 10.

The frequency of pain increases as the disease progresses, causing physical, emotional, spiritual and functional discomfort. This impedes performance of daily activities and disturbs sleeping and eating habits. As a result, cognitive function is impaired and affective, sexual and family relationships are strained, and work and leisure activities are difficult. This leads to greatly decreased QoL for these women 9.

Pain is understood as a complex multidimensional experience that must be evaluated in its affective and cognitive dimension. Emotional and cognitive factors have a strong influence on pain perception 16-18; however, untreated pain affects physical, psychological, social and spiritual well-being 19,20.

Pain is a stressful, individual and subjective human experience and has been associated with feelings of social isolation 21; thus, evaluating pain is one of the most challenging areas of care for this type of patient. The ability to accurately measure and interpret pain through valid and reliable tools or instruments may be clinically important in determining medical protocol and non-pharmaceutical interventions 22.

The purpose of this study was to evaluate the influence of pain on the QoL of women with BC. A detailed evaluation of pain enables caretakers to create a strategy to reduce pain and prevent any secondary symptoms, which improves patient QoL.

## MATERIALS AND METHODS

This prospective study was conducted with 400 women diagnosed with BC undergoing chemotherapy, radiotherapy, surgery, hormone therapy, or exclusively in palliative care. The study occurred in the oncology center at a referral hospital in a medium-sized city in the northeast of Brazil from July 2014 to April 2015. Patients were selected through non-probability sampling, and patient interviews were conducted during appointments or in the chemotherapy infusion room.

A patient was considered eligible for the study if they were diagnosed with BC, undergoing treatment for the disease or exclusively in palliative care, and were over 18. Women without cognitive ability and/or verbalization, patients who had not started treatment, and those who had been previously diagnosed with depression were excluded.

Three study groups were created within the sample: 1-118 patients without metastasis (MTX), 2- 160 patients with loco-regional MTX and 3- 122 patients with distant MTX.

Ethical issues were considered, and the Local Research Ethics Committee approved the present study (n°. CAAE 17956113.9.0000.5293) in compliance with the Declaration of Helsinki and Resolution 466/12 of the Brazilian National Health Council, which addresses research on human beings. Before starting the interview, the study objectives were explained, and the patients participating in the study were asked to sign a free and informed consent form. This ensured that participation was voluntary and that answers would be kept anonymous and confidential.

Pain was evaluated using the Short-Form McGill Pain Questionnaire (SF-MPQ) proposed by Melzack 23 and validated in Portuguese by Ferreira et al. 24. This version consists of 15 pain descriptors belonging to its three dimensions: Sensory (throbbing, jumping, flashing, sharp-pricking, tugging, burning, spreading, sore/aching), Affective (tiring-exhausting, sickening, suffocating, frightful-blinding, nagging) and Evaluative (troublesome, unbearable).

These descriptors were classified as “present” or “absent.” In addition to these descriptors, the instrument also integrates a Numeric Pain Rating Scale. The level of pain intensity was classified as mild, moderate and severe. These levels were based on the intensity with which each patient classified their pain on the Numerical Scale. Thus, 1 to 3 represents mild pain, whereas 4 to 6 represents moderate pain and 7 to 10 intense pain. The questionnaire also includes a Body Diagram to determine the location of the pain referred to by the subjects.

To assess QoL, the European Organization device for Research and Treatment for Cancer Questionnaire-Core 30 (EORTC QLQ-C30) was used. This questionnaire is a valid and reliable evaluation of cancer patient QoL and has been considered useful in many clinical trials and studies. The EORTC QLQ-C30 (version 3.0) is composed of 30 items, embedded in three scales, corresponding to the patient’s condition in the past week. The first scale, the Functional Scale, consists of five domains: physical, emotional, social, cognitive, and role functioning. The second scale, the Scale of Symptoms, consists of three sub-scales (pain, fatigue, nausea and vomiting) and six single items (dyspnea, sleep disorders, loss of appetite, constipation, diarrhea, and financial difficulties) and finally, the Global health scale.

Questions 1-28 are answered on a four-point scale, with each item classified from none (score value=1) to very much (score value=4). Questions 29 and 30 are answered on a seven-point scale going from bad (score value=1) to good (score value=7). All items were then linearly transposed onto a scale from 0 to 100. For the five functional scales and global health scale, a higher score indicates a higher level of functioning or overall QoL. Conversely, for the symptom scale and single items, a higher score implies a higher level of symptoms or problems 25.

A second questionnaire associated with the EORTC QLQ-C30, also translated and validated for use in Portuguese, was used to evaluate QoL. This instrument, EORTC BR23, is specifically used for BC patients and consists of 23 questions answered on a 4-point scale (from 1 to 4). It is composed of two scales: the Functional Scale composed of 4 sub-items (body image, sexual function, sexual pleasure and future perspectives) and the Symptom Scale consisting of 4 sub-items (side effects of systemic therapy, breast symptoms, arm symptoms and hair loss) 26. The use of these questionnaires was authorized both by Ferreira et al. 24 and the EORTC group. All procedures required by the organization were conducted. Additionally, the Karnofsky Performance Status (KPS) was used to evaluate functional capacity.

A descriptive analysis of the qualitative variables was performed through the absolute and relative frequency distribution. The T-test for independent samples was used to compare the mean total scores of the EORTC QLQ-C30 and BR23 variables for pain in the three groups of cancer patients. An analysis of variance (ANOVA) was performed to identify any existing correlations between all collected variables. Then, the Tukey test was applied to evaluate the magnitude of the latter. A significance level of 5% was used, and all calculations were performed using SPSS.V.13.

## RESULTS

Evaluating the sociodemographic profile of the 400 women, we see that most women were between 51 to 60 years of age (28.8%) or 41 to 50 (28%), and 60% were from the countryside. Other sociodemographic characteristics show that more than half, or 54.8%, had only received an elementary school education, 51% were married, only 39.3% were on sick leave, and Catholicism predominated in 63.3% of the patients. Concerning clinical variables, 87% of the women had a tumor with the histological appearance of invasive ductal carcinoma, 40% had loco-regional MTX, 76.5% had undergone some prior treatment [surgery (68.5%), chemotherapy (52.5%), radiation (22.3%), hormonal therapy (29.3%) and/or therapy with bisphosphonates (12.8%)], and 99% were undergoing some type of treatment [(surgery--late post-surgery (4%), chemotherapy (70%), radiotherapy (4%), hormonal therapy (25.3%), and/or therapy with bisphosphonates (19,8%)] at the time of the interview.

Table 1 describes the characteristics of pain, a common complaint among the women (71.7%). Pain was generally reported as diffuse in the three groups of patients. Those who reported pain in more than one site had some form of MTX (36.5% with loco-regional MTX and 45.7% with distant MTX). Interestingly, pain in the upper limbs was more prevalent in patients without MTX.

Table 1 also addresses the nature of pain, classifying it according to the frequency of sensory descriptors. For the group without MTX, words such as jumping (70.1%), sore/aching (65.7%) and sharp-pricking (61.2%) were most common. For loco-regional MTX, the subjects described the pain as jumping (80.9%), sore/aching (67.0%) and sharp-pricking (63.5%), whereas patients with distant MTX described it as sore/aching (74.3%), jumping (73.3%) and spreading (66.7%). Within the affective dimension, the most common descriptors for the group without MTX were sickening (61.2%) and nagging (59.7%). The loco-regional MTX group described it as tiring-exhausting (62.6%) and nagging (60.0%); likewise, the distant MTX group preferred words such as tiring-exhausting (80.0%) and nagging (75.2%). In the evaluative dimension, the main descriptor chosen by BC patients was “troublesome” for those without MTX (70.1%), loco-regional MTX (83.5%) and distant MTX (90.5%).

Table 2 displays the correlations found between the presence of pain and the different QoL domains (EORTC QLQ-C30 questionnaire): Women who reported pain obtained significantly different scores from those who had no complaints of pain. In the MTX-free group, the mean symptom scores for patients with pain and without pain were 29.0 (SD=15.3) and 13.4 (SD=10.8), respectively (*p*<0.001). The mean Global Health score for patients with pain was 55.3 (SD=24.8), whereas it was 69.7 (SD=19.2) for those without pain (*p*=0.001). For the loco-regional MTX group, the mean symptom score for patients with pain was 29.0 (SD=15.3), whereas those without pain had an average score of 13.4 (SD=10.8) (*p*<0.001). On the Global Health Scale, patients in pain scored 59.1 (SD=21.3), and those without pain scored 72.4 (SD=18.6) (*p*<0.001). For the group with distant MTX, the mean symptom scores for patients with pain and without pain were 39.6 (SD=19.5) and 17.5 (SD=10.0), respectively (*p*<0.001). On the Global Health Scale, the mean score for those reporting pain was 48.6 (SD=23.1), whereas the mean score for those without pain was 67.6 (SD=20.4) (*p*=0.002). It is important to highlight that the higher the score on the symptom scale, the more symptoms were listed by the patient, thus compromising their QoL.

Table 2 also shows us that the presence of pain significantly influences QoL in BC patients when evaluated by EORTC BR23. For patients with distant MTX, those who reported pain had a mean of 56.4 (SD=19.4), whereas those without pain had a mean of 68.4 (SD=13.9) (*p*=0.016). Patients without pain had a better score on self-image evaluation, thus demonstrating how pain can influence self-esteem and how the limbic system influences pain. On the Symptom Scale, the same group of patients obtained a mean of 26.9 (SD=14.2) for those who were in pain and an average of 16.5 (SD=13.8) for those without pain (*p*=0.006).

Table 3 not only displays the relationship between pain and QoL but also a possible association between pain intensity and better or worse QoL. Patients with distant MTX who rate their pain as “intense” are those with the worst scores on the QoL assessment, with a mean functional score of 49.9 (SD=17.3) (*p*<0.009), a mean symptom score of 50.0 (SD=20.1) (*p*<0.001) and a Global Health Scale mean of 39.7 (SD=24.7) (*p*<0.006). Functional capacity assessed by the KPS also showed a significant correlation between increased pain intensity and decreased QoL. The latter was observed in all three groups of patients. Women with distant MTX who reported intense pain obtained a mean KPS of 69.0 (SD=13.9) whereas mild pain reports reached a mean KPS of 78.9 (SD=8.7) (*p*<0.040).

Pain intensity was also evaluated by EORTC BR23, as shown in Table 4. Patients with distant MTX who defined their pain as “intense” scored worse on the Symptom Scale when evaluating QOL, with a mean of 32.1 (SD=14.8) (*p*=0.012). This same association was also observed in the sub-item related to “symptoms of the arm” with a mean of 40.7 (SD=29.4) (*p*=0.001).

## DISCUSSION

Pain is one of the most common and distressing symptoms experienced by cancer patients. Cancer pain involves physical, social, psychological and spiritual components, all of which belong to the term “total pain”, which is used to refer to the multidimensional nature of pain (Cicely Saunders). The contribution of each component varies based on both individual factors and circumstances faced by the patient. Therefore, the perception of pain is affected by several variables such as fatigue, insomnia, fear, anxiety, anger, sadness, depression, social isolation, altered perception of self-image and impairment of functional capacity 27.

The occurrence of pain may be due to the disease process itself or as treatment aftermath, such as post-mastectomy pain, chemotherapy-induced neuropathy or brachial plexus neuropathy from radiation therapy 28-30. This makes pain a significant problem for most women with BC, causing a negative effect on QoL.

Despite cancer pain having been very prevalent in this study (71.7% of all groups), even in the non-metastatic group (56.8), the heterogeneity of sociodemographic and clinical characteristics of the subjects did not allow a specific statistical additional comparison between groups. This result was similar to that found by Tamai et al. 31, Nabila et al. 19, and Starkweather et al. 13. Most patients in the sample had distant MTX, which agrees with the literature wherein bone, along with the lungs, is one of the major sites affected by metastatic BC 32,33. In a recent cohort study 34, bone was found to be the first site for metastatic development in 41% of the women. Bone MTX are considered the main cause of cancer pain, impairing functional capacity and limiting QoL 28,32,35.

The use of descriptors in the evaluation of pain has been increasingly considered pertinent to research and everyday clinical practice. These words help professionals determine type of pain and appropriate therapy to provide patients with the most pain relief and QoL possible 36. The SF-MPQ showed us that the descriptors most used by the studied population were sore/aching, nagging and troublesome. In agreement with our findings, in a study of 453 Norwegian cancer patients, the most commonly used descriptor for pain was aching 36. Data obtained from another study found that the most common descriptors were throbbing and tiring-exhausting 19,31,35.

When analyzing the association between the presence of pain and the EORTC domains, it was determined that pain negatively influenced QoL. This was true for the patients with and without MTX. The Global Health scale showed that women in pain considered themselves less healthy overall compared to those without pain (*p*=0.002).

The effect of pain on QoL is not only related to the intensity of pain experienced by patients but also to how pain is perceived in its multidimensional aspect 16,17,37. Our findings identified that patients who classified their pain as intense and suffered from MTX had lower mean values on the Global Health Scale. The results clearly show that the group of patients with distant MTX with acute pain was strongly associated with a decrease in QoL.

Pain seemed to impact QoL primarily in patients with distant MTX. These patients not only deal with acute pain but also have significant impairment of the Functional and Symptom Scales. They also suffer from poor body image and are emotionally scarred by the process of treatment and lack of functional capacity. The latter may be because the patient is often disfigured by the palliative treatments made necessary by advanced disease 38.

In addition, the perception of pain may contribute to a warped self-image due to the limbic system’s influence on the complexity of pain behavior. Corroborating our findings, a survey of 1965 women with BC conducted in Australia found sexual function to be reduced due to pain and negative body image 39.

Interestingly, among those without MTX, those with pain had lower means than those without for the Future Perspectives item. We believe that this reflects the fact that pain is one of the most feared symptoms, and its presence causes patients worry about its possible cause. This may lead to further worrying about the possibility of MTX and disease progression and consequently one’s mortality. The Relationship between QoL and pain seems to be inversely proportional because the higher the pain, the lower the QoL. In accordance with our results, the study by Nesvold et al. 40 identified a significant relationship between the related arm and shoulder problems of women with BC and QoL. It is interesting to note that among those without MTX, those with pain had lower means than those without pain for the Future Perspectives item. We believe that this reflects the fact that pain is one of the most feared symptoms, and its presence causes patients worry about its possible cause. This may lead to further worrying about the possibility of MTX and disease progression and consequently one’s mortality.

Despite these interesting findings, the present study should be interpreted with caution in light of its limitations. Pain was evaluated based on self-assessment and included estimates in relation to length of time, without the use of physical parameters. We recognize that some treatments by themselves can highly influence pain and QoL of these patients, and the correlation of these parameters with the results would be desirable; however, the heterogeneity of the used protocols made that impossible. Therefore, the results do not take into account the influence of confounding factors and the sample homogeneity. Further studies using different approaches with longitudinal design may provide better explanations of the complex manifestation of breast cancer.

BC is the most frequent cancer in women worldwide, and pain is one of the most common symptoms, compromising QoL. In this study, pain was highly prevalent and was detected in 71% of the patients. These findings are significant and justify more attention in the management of these patients in daily practice to minimize this unpleasant symptom that damages functional capacity and haunts the patients emotionally.

## AUTHOR CONTRIBUTIONS

Costa WA was responsible for planning the research, collecting the quantitative data, discussing the results and writing the manuscript. Monteiro MN was responsible for analyzing the qualitative data and discussing the results. Queiroz JF was responsible for analyzing the qualitative data, discussing the results and writing the manuscript. Gonçalves AK was responsible for planning and supervising the research, analyzing the data, discussing the results, producing the final revisions and submitting the manuscript.

## Figures and Tables

**Table 1:** Intensity and dimension of Pain in breast cancer patients.

Pain	Breast Cancer Patients			
Presence	Without MTX	Loco-regional MTX	Distant MTX	Total
	N (%)	N (%)	N (%)	N (%)
Yes	67 (56.8)	115 (71.9)	105 (86.1)	287 (71.7)
No	51 (43.2)	45 (28.1)	17 (13.9)	113 (28.3)
Total	118 (100.0)	160 (100.0)	122 (100.0)	400 (100.0)
Location				
Upper limb	19 (28.4)	27 (23.5)	05 (4.8)	51 (17.8)
Chest	12 (17.9)	20 (17.4)	07 (6.7)	39 (13.6)
Column	00 (0.0)	03 (2.6)	09 (8.6)	12 (4.2)
In two locations	24 (35.8)	42 (36.5)	48 (45.7)	114 (39.7)
Diffuse	12 (17.9)	23 (20.0)	36 (34.3)	71 (24.7)
Total	67 (100.0)	115 (100.0)	105 (100.0)	287(100.0)
Dimension*				
**Sensory**				
Throbbing	34 (50.7)	63 (54.8)	54 (51.4)	151 (52.6)
Jumping	47 (70.1)	93 (80.9)	77 (73.3)	217 (75.6)
Flashing	22 (32.8)	34 (29.6)	48 (45.7)	104 (29.8)
Sharp-pricking	41 (61.2)	73 (63.5)	57 (54.3)	171 (59.6)
Tugging	36 (53.7)	67 (58.3)	53 (50.5)	156 (54.3)
Burning	26 (38.8)	44 (38.3)	44 (41.9)	114 (39.7)
Spreading	27 (40.3)	50 (43.5)	70 (66.7)	147 (51.2)
Sore/aching	44 (65.7)	77 (67.0)	78 (74.3)	199 (69.3)
**Affective**				
Tiring-exhausting	37 (55.2)	72 (62.6)	84 (80.0)	187 (65.1)
Sickening	41 (61.2)	68 (59.1)	68 (64.8)	177 (61.7)
Suffocating	14 (20.9)	10 (8.7)	29 (27.6)	53 (18.5)
Frightful-blinding	14 (20.9)	14 (12.2)	27 (25.7)	55 (19.2)
Nagging	40 (59.7)	69 (60.0)	79 (75.2)	188 (65.5)
**Evaluative**				
Troublesome	47 (70.1)	96 (83.5)	95 (90.5)	238 (82.9)
Unbearable	06 (9.0)	14 (12.2)	25 (23.8)	45 (15.7)

Metastasis = MTX * Counted only for patients with pain.

**Table 2:** Correlation between the presence of Pain and Quality of Life (EORTC QLQC30/BR 23).

EORTC QLQC30	Presence of pain				*p*-value
	Yes		No		
	Mean	SD	Mean	SD	
	Without MTX				
Functional Scales	59.8	19.8	76.9	14.7	<0.001*
Physical functioning	69.9	21.4	84.4	16.0	<0.001*
Role functioning	37.1	32.0	60.4	32.3	<0.001*
Cognitive functioning	65.9	31.8	81.7	20.1	0.001*
Emotional functioning	47.0	29.3	65.7	25.6	<0.001*
Social functioning	77.1	32.8	91.8	19.2	0.003*
Symptom Scales	29.0	15.3	13.4	10.8	<0.001*
Global Health Scales	55.3	24.8	69.7	19.2	0.001*
	Loco-regional MTX				
Functional Scales	63.9	17.5	74.7	15.4	<0.001*
Physical functioning	69.7	21.4	78.5	22.4	0.023*
Role functioning	38.8	30.1	53.7	34.2	0.008*
Cognitive functioning	75.5	26.1	83.0	19.9	0.055
Emotional functioning	55.6	29.1	68.5	25.4	0.010*
Social functioning	79.8	28.7	89.2	18.8	0.017*
Symptom Scales	29.0	15.3	13.4	10.8	<0.001*
Global Health Scales	59.1	21.3	72.4	18.6	<0.001*
	Distant MTX				
Functional Scales	54.1	17.4	73.1	17.5	<0.001*
Physical functioning	54.0	27.4	74.5	23.9	0.004*
Role functioning	27.1	31.3	53.9	36.1	0.002*
Cognitive functioning	70.5	30.2	79.4	23.9	0.249
Emotional functioning	47.9	27.7	70.1	29.0	0.003*
Social functioning	77.1	29.6	88.2	20.2	0.061
Symptom Scales	39.6	19.5	17.5	10.0	<0.001*
Global Health Scales	48.6	23.1	67.6	20.4	0.002*
**EORTC BR23**					
	Without MTX				
Functional Scales	56.6	20.2	67.8	15.7	0.001*
Body image	68.8	30.9	84.3	22.1	0.002*
Sexual functioning	26.1	27.9	27.8	28.8	0.753
Sexual enjoyment	51.0	32.8	58.7	31.4	0.400
Future perspective	31.3	37.1	47.1	39.5	0.029*
Symptom Scales	30.9	18.5	13.0	13.0	<0.001*
	Loco-regional MTX				
Functional Scales	61.1	17.4	71.0	12.0	<0.001*
Body image	73.5	27.5	87.0	17.4	<0.001*
Sexual functioning	29.1	29.5	21.8	28.6	0.159
Sexual enjoyment	59.0	35.4	56.4	28.5	0.812
Future perspective	39.7	39.5	65.2	41.0	<0.001*
Symptom Scales	29.0	15.3	13.4	10.8	<0.001*
	Distant MTX				
Functional Scales	56.4	19.4	68.4	13.9	0.016*
Body image	68.2	32.7	86.3	18.8	0.003*
Sexual functioning	23.8	26.0	26.5	27.0	0.697
Sexual enjoyment	47.9	30.6	62.5	21.4	0.203
Future perspective	36.2	39.3	49.0	33.6	0.205
Symptom Scales	26.9	14.2	16.5	13.8	0.006*

Metastasis = MTX * T-test.

**Table 3:** Correlation between the level of Pain, Quality of Life and Functional capacity.

EORTC QLQ30/KPS	Intensity of pain						*p*-value
	Mild		Moderate		Intense		
	Mean	SD	Mean	SD	Mean	SD	
	Without MTX						
Functional Scales	69.3a	16.6	54.0b	18.6	50.3b	22.0	0.003*
Physical functioning	81.2a	13.7	64.0b	23.4	56.4b	19.8	<0.001*
Role functioning	43.8	29.3	32.1	32.7	33.3	36.5	0.370
Cognitive functioning	73.2	30.9	58.9	31.2	65.1	34.5	0.246
Emotional functioning	58.9a	31.2	40.2b	25.7	34.1b	23.7	0.014*
Social functioning	83.3	29.0	73.8	35.3	69.7	35.6	0.402
Symptom Scales	22.1a	15.2	30.3ab	16.8	39.4b	15.1	0.009*
Global Health Scales	60.1	21.8	51.8	24.1	52.3	32.9	0.416
KPS	92.9a	6.6	86.1b	10.7	80.0b	13.4	0.001*
	Loco-regional MTX						
Functional Scales	24.1	13.3	73.6	14.5	38.2	14.8	0.321
Physical functioning	73.6	22.1	67.5	21.1	66.7	21.1	0.295
Role functioning	40.2	30.3	41.7	32.5	30.3	21.8	0.249
Cognitive functioning	74.0	27.4	80.1	23.7	70.2	27.7	0.264
Emotional functioning	57.9	28.9	58.3	28.2	47.3	29.7	0.228
Social functioning	80.5	27.9	75.7	31.0	85.7	25.9	0.347
Symptom Scales	27.5a	13.9	33.6ab	14.3	23.5b	16.8	0.015*
Global Health Scales	59.3	20.8	60.1	21.9	55.9	20.3	0.698
KPS	88.5a	7.3	87.4a	9.7	82.5b	8.8	0.013*
	Distant MTX						
Functional Scales	61.7a	15.4	56.1ab	16.8	47.9b	17.3	0.009*
Physical functioning	65.6a	22.7	56.3ab	26.1	45.6b	29.0	0.024*
Role functioning	39.5	33.9	56.3	26.1	19.2	28.0	0.061
Cognitive functioning	76.3	25.0	65.6	32.7	73.5	29.3	0.317
Emotional functioning	53.5	31.2	52.8	25.5	39.3	26.9	0.048
Social functioning	76.3	30.1	80.1	24.7	73.9	34.8	0.625
Symptom Scales	23.6a	10.5	37.5b	16.8	50.0c	20.1	<0.001*
Global Health Scales	50.0a	20.2	55.5ab	20.6	39.7b	24.7	0.006*
KPS	78.9a	8.7	75.5a	69.0	69.0b	13.9	0.040*

Metastasis = MTX * ANOVA - test.

a,ab,b: Tukey test - the means of the different letters are statistically significant.

**Table 4:** Correlation between the level of Pain and Quality of Life in breast cancer women.

EORTC BR23	Intensity of pain						*p*-value
	Mild		Moderate		Intense		
	Mean	SD	Mean	SD	Mean	SD	
	Without MTX						
Symptom Scales	26.1	17.7	33.2	17.8	37.6	20.6	0.154
Systemic therapy side effects	30.3	24.1	35.9	25.9	39.8	21.8	0.495
Breast symptoms	32.4	18.3	34.8	20.8	32.6	29.9	0.908
Arm symptoms	14.7a	18.4	31.7a	25.4	45.5b	21.3	<0.001*
Upset by hair loss	5.9	43.6	11.9	53.4	18.2	58.4	0.779
	Loco-regional MTX						
Symptom Scales	30.7	14.8	32.3	15.0	26.3	13.5	0.225
Systemic therapy side effects	31.0	18.4	38.0	23.4	26.5	21.0	0.073
Breast symptoms	39.0	20.2	30.8	19.5	32.1	19.2	0.145
Arm symptoms	27.0	20.0	26.1	19.8	26.0	19.6	0.974
Upset by hair loss	12.2	22.1	12.3	25.7	7.1	18.9	0.591
	Distant MTX						
Symptom Scales	22.6a	10.8	24.3b	13.8	32.1b	14.8	0.012*
Systemic therapy side effects	24.8	14.5	27.9	19.9	36.1	18.5	0.046
Breast symptoms	27.2	14.4	27.7	19.2	29.1	27.4	0.939
Arm symptoms	22.2a	22.2	21.7b	17.9	40.7b	29.4	0.001*
Upset by hair loss	10.5	31.5	7.1	42.2	10.3	42.7	0.920

Metastasis = MTX * ANOVA -test.

a,ab,b: Tukey test - the means of the different letters are statistically significant.

## References

[1] Ferly J, Soerjomataram I, Dikshit R, Eser S, Mathers C, Rebelo M (2015). Cancer incidence and mortality worldwide: sources, methods and major patterns in GLOBOCAN 2012. Int J Cancer.

[2] Hsu HT, Lin KC, Wu LM, Juan CH, Hou MF, Hwang SL (2017). Symptom Cluster trajectories during chemotherapy in breast cancer outpatients. J Pain Symptom Manage.

[3] Lee YP, Wu CH, Chiu TY, Chen CY, Morita T, Hung SH (2015). The relationship between pain management and psychospiritual distress in patients with advanced cancer following admission to a palliative care unit. BMC Palliat Care.

[4] Liamputtong P, Suwankhong D (2015). Breast cancer diagnosis: biographical disruption, emotional experiences and strategic management in Thai women with breast cancer. Sociol Health IIIn.

[5] Lemay K, Wilson KG, Buenger U, Jarvis V, Fitzgibbbon E, Bhimji K (2011). Fear of pain in patients with advanced cancer or in patients with chronic noncancer pain. Clin J Pain.

[6] Langford DJ, Schmidt B, Levine JD, Abrams G, Elboim C, Esserman L (2015). Preoperative breast pain predicts persistent breast pain and disability after breast cancer surgery. J Pain Symptom Manage.

[7] Langford DJ, Paul SM, Cooper B, Kober KM, Mastick J, Melisko M (2016). Comparison of subgroups of breast cancer patients on pain and co-occurring symptoms following chemotherapy. Support Care Cancer.

[8] Didwaniya N, Tanco K, de la Cruz M, Bruera E (2015). The need for a multidisciplinary approach to pain management in advanced cancer: a clinical case. Palliat Support Care.

[9] Hui D, Bruera E (2014). A personalized approach to assessing and managing pain patients with cancer. J. Clin. Oncol.

[10] van den Beuken-van Everdingen MH, Hochstenbach LM, Joosten EA, Tjan-Heijnen VC, Janssen DJ (2016). Update on prevalence of pain in patients with cancer: Systematic Review and Meta-Analysis. J Pain Symptom Manage.

[11] Variawa ML, Scribante J, Perrie H, Chetty S (2016). The prevalence of chronic postmastectomy pain syndrome in female breast cancer survivors. South Afr J Anaesth Analg.

[12] Song SJ, Min J, Suh SY, Jung SH, Hahn HJ, Im SA (2017). Incidence of taxane-induced peripheral neuropathy receiving treatment and prescription patterns in patients with breast cancer. Support Care Cancer.

[13] Starkweather AR, Lyon DE, Schubert CM (2013). Pain and inflammation in women with early-stage breast cancer prior to induction of chemotherapy. Biol Res Nurs.

[14] Juhl AA, Christiansen P, Damsgaard TE (2016). Persistent pain after breast cancer treatment: a Questionnaire-Based Study on the prevalence, associated treatment variables, and pain type. J Breast Cancer.

[15] Miaskowski C, Cooper BA, Paul SM, Dodd M, Lee K, Aouizerat BE (2006). Subgroups of patients with cancer with different symptom experiences and quality-of-life outcomes: a cluster analysis. Oncol Nurs Forum.

[16] Johannsen M, O’Connor M, O’Toole MS, Jensen AB, Hojris I, Zachariae R (2016). Efficacy of mindfulness-based cognitive therapy on late post-treatment pain in women treated for primary breast cancer: a randomized controlled trial. J Clin Oncol.

[17] Bushenell MC, Ceko M, Low LA (2013). Cognitive and emotional control of pain and its disruption in chronic pain. Nat. Rev. Neurosci.

[18] IASP (2007). International Association for the Study of Pain, IASP Taxonomy (term: pain).

[19] Rouahi N, Ouazzani Touhami Z, Ahyayauch H, El Mlili N, Filali-Maltouf A, Belkhadir Z (2016). Assessment of the Nature and severity of pain using SF-MPQ for cancer patients at the National Institute of Oncology in Rabat in 2015. Asian Pac J Cancer Prev.

[20] Delgado-Guay MO, Hui D, Parsons HA, Govan K, De la Cruz M, Thorney S (2011). Spirituality, religiosity, and spiritual pain in advanced cancer patients. J Pain Symptom Manage.

[21] de Papathanassoglou E (2014). Recent advances in understanding pain: what lies ahead for critical care. Nurs Crit Care.

[22] Johnson AM, Smith SM (2017). A review of general pain measurement tools and instruments for consideration of use in COPD clinical practice. Int J Chron Obstruct Pulmon Dis.

[23] Melzack R (1987). The short-form McGill Pain Questionnaire. Pain.

[24] Ferreira KA, de Andrade DC, Teixeira MJ (2013). Development and validation of a Brazilian version of short-form McGill pain questionnaire (SF-MPQ). Pain Manag Nurs.

[25] Aaronson NK, Ahmedzai S, Bergman B, Bullinger M, Cull A, Duez NJ (1993). The European Organization for Research and Treatment of Cancer QLQ-C30: a quality-of-life instrument for use in international clinical trials in oncology. J Natl Cancer Inst.

[26] Sprangers MA, Groenvold M, Arraras JI, Franklin J, te Velde A, Muller M (1996). The European Organization for Research and Treatment of Cancer breast cancer-specific quality-of-life questionnaire module: first results from a three-country field study. J Clin Oncol.

[27] Mehta A, Chan LS (2008). Understanding of the concept of &ldquo;Total Pain&rdquo; A prerequisite for pain control. Journal of Hospice and Palliative Nursing.

[28] Leppert W, Zajaczkowska R, Wordliczek J, Dobrogowski J, Woron J, Krzakowski M (2016). Pathophysiology and clinical characteristics of pain in most common locations in cancer patients. J Physiol Pharmacol.

[29] Leclerc AF, Jerusalem G, Devos M, Crielaard JM, Maquet D (2016). Multidisciplinary management of breast cancer. Arch Public Health.

[30] Seretny M, Currie GL, Sena ES, Ramnarine S, Grant R, MacLeod MR (2014). Incidence, prevalence, and predictors of chemotherapy-induced peripheral neuropathy: a systematic review and meta-analysis. Pain.

[31] Tamai N, Mugita Y, Ikeda M, Sanada H (2016). The relationship between malignant wound status and pain in breast cancer patients. Eur J Oncol Nurs.

[32] Fontanella C, Fanotto V, Rihawi K, Aprile G, Puglisi F (2015). Skeletal metastases from breast cancer: pathogenesis of bone tropism and treatment strategy. Clin Exp Metastasis.

[33] Piccioli A, Maccauro G, Spinelli MS, Biagini R, Rossi B (2015). Bone metastases of unknown origin: epidemiology and principles of management. J Orthop Traumatol.

[34] Berman AT, Thukral AD, Hwang WT, Solin LJ, Vapiwala N (2013). Incidence and patterns of distant metastases for patients with early-stage breast cancer after breast conservation treatment. Clin. Breast Cancer.

[35] Shibata H, Kato S, Sekine I, Abe K, Iguchi H, Izumi T (2016). Diagnosis and treatment of bone metastasis: comprehensive guideline of the Japanese Society of Medical Oncology, Japanese Orthopedic Association, Japanese Urological Association, and Japanese Society for Radiation Oncology. ESMO Open.

[36] Holtan A, Kongsgaard UE (2009). The use of pain descriptors in cancer patients. J Pain Symptom Manage.

[37] Lamé IE, Peters ML, Vlaeyen JW, Kleef Mv, Patijn J (2005). Quality of life in chronic pain is more associated with beliefs about pain, than with pain intensity. Eur J Pain.

[38] Chen CL, Liao MN, Chen SC, Chan PL, Chen SC (2012). Body image and its predictors in breast cancer patients receiving surgery. Cancer Nurs.

[39] Ussher JM, Perz J, Gilbert E (2012). Changes to sexual well-being and intimacy after breast cancer. Cancer Nurs.

[40] Nesvold IL, Reinertsen KV, Fossa SD, Dahl AA (2011). The relation between arm/shoulder problems and quality of life in breast cancer survivors: a cross-sectional and longitudinal study. J Cancer Surviv.

